# Targeting Chitinase 1 and Chitinase 3-Like 1 as Novel Therapeutic Strategy of Pulmonary Fibrosis

**DOI:** 10.3389/fphar.2022.826471

**Published:** 2022-03-17

**Authors:** Suh-Young Lee, Chang-Min Lee, Bing Ma, Suchitra Kamle, Jack A. Elias, Yang Zhou, Chun Geun Lee

**Affiliations:** ^1^ Molecular Microbiology and Immunology, Brown University, 185 Meeting St., Providence, RI, United States; ^2^ Devision of Allergy and Clinical Immunology, Department of Internal Medicine, Seoul National University Hospital, Seoul, South Korea

**Keywords:** chitinase 1, chitinase-like proteins, chitinase 3-like 1, pulmonary fibrosis, foxo3, tgfbrap1

## Abstract

Chitinase 1 (CHIT1) and chitinase 3-like-1 (CHI3L1), two representative members of 18-Glycosyl hydrolases family, are significantly implicated in the pathogenesis of various human diseases characterized by inflammation and remodeling. Notably, dysregulated expression of CHIT1 and CHI3L1 was noted in the patients with pulmonary fibrosis and their levels were inversely correlated with clinical outcome of the patients. CHIT1 and CHI3L1, mainly expressed in alveolar macrophages, regulate profibrotic macrophage activation, fibroblast proliferation and myofibroblast transformation, and TGF-β signaling and effector function. Although the mechanism or the pathways that CHIT1 and CHI3L1 use to regulate pulmonary fibrosis have not been fully understood yet, these studies identify CHIT1 and CHI3L1 as significant modulators of fibroproliferative responses leading to persistent and progressive pulmonary fibrosis. These studies suggest a possibility that CHIT1 and CHI3L1 could be reasonable therapeutic targets to intervene or reverse established pulmonary fibrosis. In this review, we will discuss specific roles and regulatory mechanisms of CHIT1 and CHI3L1 in profibrotic cell and tissue responses as novel therapeutic targets of pulmonary fibrosis.

## Introduction

Pulmonary fibrosis is a devastating lung disease that affects up to 200,000 people in the US alone. Idiopathic Pulmonary Fibrosis (IPF) is the most common form of pulmonary fibrosis and has a poor life expectancy (2 to 3 years of median survival) after diagnosis (Michaelson et al., 2000; Ley et al., 2011). Currently, there are two FDA approved therapeutic drugs for PF: pirfenidone (Esbriet^®^) and nintedanib (Ofev or Vargatef^®^). These drugs provide substantial benefits by slowing disease progression, but they do not relive symptom or improve quality of life and are associated with severe side effects and prohibitively high cost. Additionally, available treatments do not reverse lung damage incurred during fibrotic progression, necessitating early treatments prior to the destruction of normal lung architecture. The only curative treatment for PF is a high-risk lung transplant and life-long anti-rejection treatments. However, lung transplants are associated with extreme morbidity and recipients often succumb to fibrotic rejection within 5–10 years of the transplant. Therefore, new drug or methods of prevention and treatment of PF are critically needed.

IPF is a progressive lung disease characterized by epithelial damage, fibroproliferative matrix deposition and parenchymal remodeling (Raghu, 1998; Selman et al., 2001; Krein and Winston, 2002). Although extensive epidemiological studies suggested that environmental and occupational exposure to noxious materials or particulate matters such as silica, air pollution, cigarette smoke or certain drugs cause fibrosis, the exact etiology of PF has largely remained “idiopathic”. TGF-β1 is believed to play an important role in this dysregulation because it is expressed in an exaggerated fashion in IPF where, in contrast to controls, a sizable percentage is biologically active (Khalil et al., 1996; Khalil et al., 2001; Xu et al., 2003). The important role that TGF-β1 may play in this disorder can be seen in studies that demonstrate that TGF-β1 is a critical mediator of pulmonary fibrosis after bleomycin injury (Nakao et al., 1999; Yehualaeshet et al., 2000) and that high dose adenoviral TGF-β1 transfer or lung-specific transgenic expression causes progressive pulmonary fibrosis *in vivo* (Sime et al., 1997; Kelly et al., 2003; Lee et al., 2004) and IPF-like fibroblastic foci *in vitro* explants (Xu et al., 2003). However, the factors that control these TGF-β1 responses that allow TGF-β1 to contribute to the pathogenesis of pulmonary fibrosis are still poorly understood.

Recent studies identified that chitinase 1 (CHIT1) and chitinase 3-like-1 (CHI3L1), two representative members of 18-Glycosyl hydrolase (18-GH) family, are significantly implicated in the pathogenesis of various human diseases characterized by inflammation and remodeling (Kanneganti et al., 2012; Hong et al., 2018; Zhao et al., 2020). Dysregulated expression of CHIT1 and CHI3L1 was noted in the patients with pulmonary fibrosis and their levels were inversely correlated with clinical outcome of the patients (Lee et al., 2012; Zhou et al., 2014). The macrophages are the major cells expressing CHIT1 and CHI3L1, and *in vitro* and *in vivo* studies demonstrated their regulatory roles in profibrotic macrophage activation, fibroblast proliferation, myofibroblast transformation, and TGF-β signaling and effector function (Lee et al., 2012; Zhou et al., 2014; Zhou et al., 2015; Lee et al., 2019). Preclinical studies using animal model of pulmonary fibrosis further demonstrated that both CHIT1 and CHI3L1 are sufficient and required for fibroproliferative responses. These studies suggest a possibility of re-programing of these profibrotic cells to reverse established pulmonary fibrosis by intervention of CHIT1 and or CHI3L1. Here we will overview the recent progress that revealed a new paradigm on the pathogenesis of pulmonary fibrosis and will discuss a new therapeutic strategy by targeting both CHIT1 and CHI3L1.

### Current Therapeutics Being Used in the Patients With Pulmonary Fibrosis

Most clinical trials have been conducted on the patients with IPF based international multidisciplinary consensus classification of idiopathic interstitial pneumonias (Demedts and Costabel, 2002). Since the role of specific inflammatory cells in the pathogenesis of fibrosis is controversial (Gauldie, 2002; Strieter, 2002), anti-inflammatory agents represented by corticosteroids did not show a significant effect in the treatment of IPF (Richeldi et al., 2003). In addition, considering the risk of potential side effects, long-term, systemic use of high-dose anti-inflammatory drugs are not recommended. Even with combinatorial approaches using multiple drugs, prednisone, azathioprine, and N-acetylcysteine (NAC), rather increased the risk of death and hospitalization in IPF patients compared to the patients with placebo (Idiopathic Pulmonary Fibrosis Clinical Research et al., 2012). Anticoagulant warfarin did not show a benefit in the treatment of patients with progressive IPF (Noth et al., 2012). Ambrisentan, a selective endothelin receptor antagonist, was not effective in treating IPF and may be associated with an increased risk for disease progression and respiratory hospitalizations (Raghu et al., 2013). Imatinib, a serine/threonine kinase inhibitor, also did not affect survival or lung function (Daniels et al., 2010). Thus, these drugs either in single or combinational uses are not strongly recommended for the patients with IPF in the current guidelines (Raghu et al., 2015). The use of phosphodiesterase-5 inhibitor (Sildenafil) and dual endothelin receptor antagonists (Macitenta and Bosentan) were considered to use in certain cases of IPF patients with pulmonary arterial hypertension, but recent clinical studies did not show significant benefits of these drugs to these patients (Lee and Song, 2020; Kang and Song, 2021).

The currently recommended FDA-approved drugs for IPF are pirfenidone and nintedanib. Both pirfenidone and nintedanib have been shown to slow the lung function deterioration. Pirfenidone is a small synthetic molecule with antifibrotic properties. It reduce the expression of TGF-β in the lung and inhibits the recruitment of fibrocytes to the lung in bleomycin-induced lung fibrosis model (Myllärniemi and Kaarteenaho, 2015). In a phase II study, significantly reduced loss of VC was demonstrated in patients with pirfenidone (Taniguchi et al., 2010). Following phase III studies showed a beneficial effect on reduction in decline of FVC for pirfenidone compared to placebo (Noble et al., 2011; King et al., 2014). Nintedanib is an inhibitor of the *Src* family of tyrosine kinases, that inhibits VEGF and PDGF receptors (Myllärniemi and Kaarteenaho, 2015). Nintedanib inhibited proliferation, migration, and transformation of fibroblasts to myofibroblasts (Wollin et al., 2015). In two replicate phase 3 trials, nintedanib reduced the decline in FVC, which is consistent with a slowing of disease progression (Richeldi et al., 2014). There is no head-to-head comparison study performed between pirfenidone and nintedanib. Since currently available antifibrotic drugs cannot control or reverse the disease status, but only slow the loss of lung function, there is a strong need for effective, and less-toxic therapeutic methods available for the patients with pulmonary fibrosis.

### Major Cellular and Tissue Factors Implicated in Pathogenesis of Pulmonary Fibrosis

Tissue fibrosis is a major cause of morbidity in pulmonary fibrosis. As a normal repair response, fibrosis is a series of process of cellular damage caused by various conditions that initiate inflammation, recruitments of inflammatory cells, followed by final tissue repair and termination of inflammation. Loss of regulatory signals and imbalance in the process of wound healing leads to aberrant activation of repair response, causing pathologic fibrosis in various organs including lung, resulting in a disease state (Wilson and Wynn, 2009). Since excellent review articles detailing the molecules and signaling pathways involved in the pathogenesis of pulmonary fibrosis are already available (Micallef et al., 2012; Wolters et al., 2014; Sgalla et al., 2018; Strykowski and Adegunsoye, 2021), in this section, we only focus on the discussion of major factors leading to pathologic fibrosis, that can be also regulated by CHIT1 or CHI3L1 in the development and progression of pulmonary fibrosis. Pulmonary fibrosis comprises a number of different etiologies and pathologies with completely different clinical features and therapeutic responses. Accordingly, there are significant limitations in identifying common pathogenetic mechanisms of pulmonary fibrosis. This is particularly true for *in vitro* cell or *in vivo* preclinical animal models of pulmonary fibrosis, since currently no preclinical models are exactly representing the characteristic cellular and tissue responses of IPF and other interstitial lung disease (ILD). In addition, so far relatively small number of human studies with dysregulated expression of CHIT1 and/or CHI3L1 in the patients with IPF and ILD add certain limitations in direct clinical translation of preclinical data. With these limitations in mind, here the molecular and mechanistic implications of CHIT1 and CHI3L1 as potential therapeutic targets are discussed based on up-to-dated and common lung pathologies of pulmonary fibrosis.

#### Myofibroblasts

Compared to normal wound healing, excessive accumulation of myofibroblasts plays a key role in fibrotic tissue responses. Activated myofibroblasts expressing α-smooth muscle actin (SMA), collagens and other extracellular matrix noted at sites of fibrotic foci are one of the pathologic hallmarks of pulmonary fibrosis. Although multiple types of cells which include resident interstitial fibroblasts, circulating fibrocytes or progenitor cells, epithelial cells and endothelial pericytes have been thought as sources of myofibroblasts (Zent and Guo, 2018; Hung, 2020), the exact origin of myofibroblasts in fibrotic foci is still largely elusive. Recent single cell RNAseq analysis identified different subsets of fibroblasts which have distinct expression profile and nature in the development of fibrotic cellular and tissue responses (Tsukui et al., 2020). The fibroblasts isolated from the lungs of progressive IPF with expression of PD-L1 or specific integrin receptors, such as αvβ6, demonstrated higher invasiveness compared to normal lung fibroblasts (Chen et al., 2016; Geng et al., 2019). Thus, understanding the exact origin, differentiation, and activation mechanism(s) of invasive fibroblasts/myofibroblasts would be essential for the development of effective therapeutics against pulmonary fibrosis with invasive and progressive nature. While the myofibroblasts noted in the normal repair process are known to be removed from the sites of normal wound healing through apoptotic cell death response, the invasive myofibroblasts at site of pathologic fibrosis are resistant to dying out (Hinz and Lagares, 2020). Thus, either de-differentiate of the invasive myofibroblasts to normal fibroblasts through reprogramming or the induction of apoptosis of myofibroblasts could be a reasonable therapeutic strategy to reverse established fibrosis in the patients with IPF.

#### Profibrotic Macrophages

In addition to myofibroblasts, recent studies also revealed a new regulatory role of inflammatory and immune cells in the development and resolution of pathologic fibrosis. Notably, specific subset of macrophages called as “profibrotic macrophages” were identified in the lungs of IPF patients as well as in the animal models of pulmonary fibrosis (Misharin et al., 2017; Aran et al., 2019; Ayaub et al., 2021). These macrophages, originated from circulating monocytes but not form tissue resident macrophages, contribute to fibrotic tissue response through expression of profibrotic growth factors and cytokines as well as extracellular matrix proteins. Recent *in vivo* single cell and bulk RNA sequencing analysis revealed impressive phenotype changes in the development of profibrotic macrophages from the circulating monocytes to interstitial and resident alveolar macrophages according to the progress of fibrosis (Misharin et al., 2017; Aran et al., 2019; Ayaub et al., 2021). These studies strongly suggest that profibrotic macrophages play a critical role not only in the initiation but also progression and resolution of pathologic fibrosis. The impressive plasticity of the macrophages also supports a possibility of re-programming of the macrophages to reverse profibrotic to non-profibrotic macrophages as a therapeutic strategy of pulmonary fibrosis. Recent studies identified that Bcl2 coupled with Carnitine palmitoyltransferase 1a (Cpt1a), the mitochondrial rate-limiting enzyme for fatty acid β-oxidation, confers apoptosis resistance of macrophages at site of pathologic fibrosis and dysregulated fibrotic remodeling (Gu et al., 2021). Thus, further identification of the factors or the pathways regulating the recruitment, activation and clearance of profibrotic macrophages will be essential to understand the process of pathologic fibrosis as well as for the development of therapeutics to reverse established progressive pulmonary fibrosis.

#### Collagens and Extracellular Matrix

The pathologic hallmark of pulmonary fibrosis is excessive accumulation of various types of collagens and other extracellular matrix proteins. Traditionally, collagen accumulation at sites of pathologic fibrosis is considered as a result of imbalance between newly generated collagens vs. collagen degradation. On this regard, the prevailing hypothesis was that either deficiencies of collagen degrading enzyme matrix metalloproteinases (MMPs) or the excess of tissue inhibitors of metalloproteinases (TIMPs), that result into the dysregulated collagen accumulation (Pardo et al., 2016). However, in contrast to our expectation, recent preclinical studies using targeted null mutant mice demonstrated that MMP-9 or MMP-12 did not show significant impact on the bleomycin induced pulmonary fibrosis (Craig et al., 2015). In addition, TIMP1 null mutant mice also did not reduce collagen accumulation in the lungs of animal models of pulmonary fibrosis (Kim et al., 2005). These studies suggest that other intrinsic and extrinsic factors are implicated in the regulation of collagen accumulation in the lung. Recent studies brought attention to the increased collagen stability at sites of pathologic fibrosis compared to normal wound healing. The deficiency of Lysyl oxidase (LOX) or Lysyl Oxidase-like Proteins (LOX/Ls) significantly reduced collagen accumulation in lungs of murine model of pulmonary fibrosis, since processed collagen (cross-linked collagen) could not be efficiently degraded by collagen degrading enzymes (Tjin et al., 2017; Bellaye et al., 2018). Collagens with certain epigenetic changes were noted only in the lungs of IPF patients, further support this notion that aberrant collagens are generated and accumulated in the lungs of pulmonary fibrosis (Merl-Pham et al., 2019). The expression and the stability of the collagens can be also regulated by transcriptional factors or microRNA, such as mir29, that directly binds to collagen RNAs or other mechanism of posttranscriptional modifications (Yano et al., 2018). These studies further suggest a possibility that collagen modifying enzymes or interacting proteins contribute to the excessive accumulation of collagens in the lungs of patients with pulmonary fibrosis.

#### TGF-β Expression and Signaling

Many signaling pathways that regulate cell differentiation, migration, and transition are implicated in the pathology of pulmonary fibrosis (Chanda et al., 2019). Among these, TGF-β signaling pathway has long been considered as a key player in initiation and progression of pulmonary fibrosis (Yue et al., 2010). TGF-β induces epithelial or endothelial mesenchymal transition (EMT and EndoMT), fibroblast proliferation and myofibroblast transformation through canonical and noncanonical signaling molecules such as Smads, PI3K-AKT, and MAPK (Kasai et al., 2005; Kim et al., 2006; Hashimoto et al., 2010). TGF-β also known to play a significant role in resolution of fibrosis in the process of normal healing by inducing apoptosis of cells responsible for pathologic fibrosis (Müller et al., 1997; Tschopp et al., 1998). However, factors or pathways regulating effector function of TGF-β between normal repair vs. pathologic fibrosis are not still clearly understood.

### Role of CHIT1 in the Pathogenesis of Pulmonary Fibrosis

Although mammals do not have chitin or chitin synthase, substantial levels of chitinases and chitinase-like proteins (CLP) are noted in the circulation as well as in local tissues (Lee et al., 2009; Lee et al., 2011; Cho et al., 2015). CHIT1, a major enzymatically active true chitinase, is produced, stored, and secreted by macrophages and neutrophils (van Eijk et al., 2005) and plays important roles in innate immune homeostasis (Elias et al., 2005). This can be appreciated in the pivotal roles it plays in host defenses against chitin-containing pathogens such as fungi, protozoa and insects (Boot et al., 2001). As a sensitive biomarker of macrophage activation, dysregulated expression of CHIT1 in the circulation or local tissue has been reported in a variety of human diseases including Gaucher’s disease, diabetes, sarcoidosis, inflammatory bowel disease, atherosclerosis, Alzheimer’s disease, NASH and prostate cancer (Kanneganti et al., 2012; Elmonem et al., 2016). Recent studies also demonstrated that CHIT1 is dysregulated in lung diseases characterized by inflammation and remodeling such as bacterial infection, asthma, COPD and pulmonary fibrosis (Lee et al., 2012; Cho et al., 2015; James et al., 2016; Hong et al., 2018; Sharma et al., 2018; Lee et al., 2019). Recent human and preclinical studies demonstrated that CHIT1 plays an important role in the pathogenesis of both IPF and scleroderma-associated interstitial lung disease (SSc-ILD) (Lee et al., 2012; Lee et al., 2019). These studies further identified specific signaling pathways and interacting partners that CHIT1 uses to contribute to the pathogenesis of pulmonary fibrosis.

#### CHIT1 Expression in Human Lung Fibrosis

**FIGURE 1:**
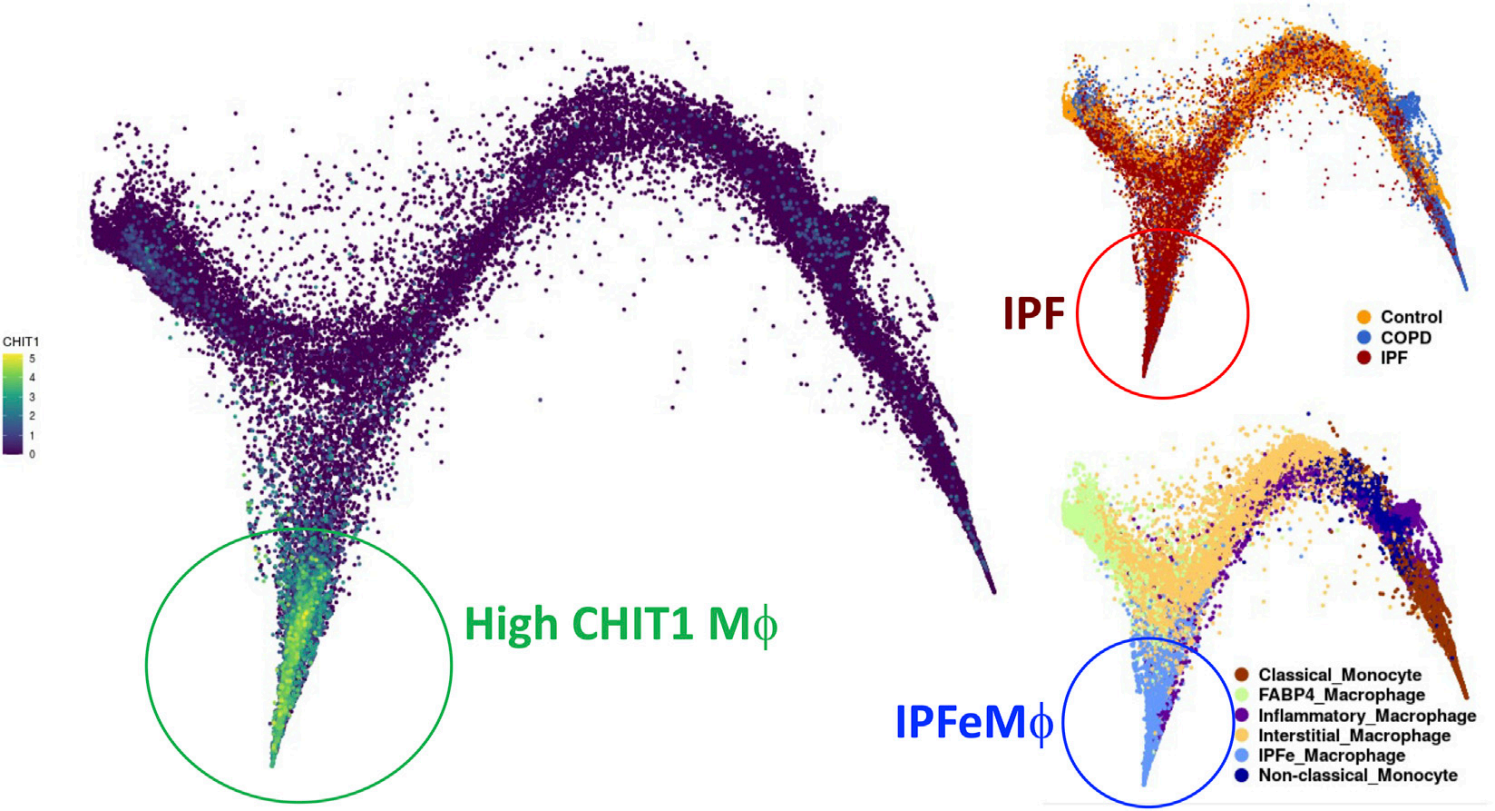
UMAP (uniform manifold approximation and projection) clustering of lung macrophages in patients with IPF evaluated by single cell RNAseq analysis. Highly CHIT1 expressing macrophages (green, left) are overlapping with macrophage subset of patients with IPF (dark brown, upper right) but not with control or COPD patients (blue or bright brown, right upper). It is also notable that most of these CHIT1 expressing macrophages are significantly overlapping with IPF expanded macrophages (IPFeMϕ) (Blue, right lower), the distinct subset of profibrotic macrophages noted in IPF patients. Publicly available RNAseq data (http://www.ipfcellatlas.com/; Single cell RNA-seq, 2021, Ivan Rosas group) was re-plotted for CHIT1 expressing macrophages.

Significant increases in the expression of CHIT1 were noted in lungs of the patients with SSc-ILD as well as in the IPF patients (Lee et al., 2012; Lee et al., 2019). The increased levels of CHIT1 activities in the serum of the patients with SSc-ILD and they are inversely correlated with lung function and overall survival (Lee et al., 2012; Lee et al., 2019). Immunohistochemistry (IHC) analysis localized macrophages as the major cells expressing CHIT1. Modestly increased expression of CHIT1 was also noted in epithelial cells and other interstitial parenchymal cells in the lungs of IPF patients. Interestingly, single cell RNASeq transcriptome analysis on multiple cohorts of IPF patients identified subset of macrophages that highly express CHIT1 and that distinctly overlaps with macrophage populations from the patients with IPF but not with normal controls or COPD patients (Figure 1). These studies suggest a significant implication of CHIT1 in the pathogenesis of pulmonary fibrosis as a factor of macrophage differentiation.

#### CHIT1 in Profibrotic Macrophage Differentiation

As discussed above, profibrotic macrophage activation significantly contribute to the development of pulmonary fibrosis (Wynn and Vannella, 2016; Lee et al., 2018). Since the macrophages are the major cells expressing CHIT1 in IPF lungs, it is reasonable to assume that CHIT1 could plays an important role in profibrotic macrophage differentiation. In support of this notion, *in vitro* macrophages differentiation studies using alveolar macrophages isolated from wild type and CHIT1 null mutant mice, demonstrated that CHIT1 plays a critical role in recombinant (r) IL-4 or rTGF-β stimulated fibrotic macrophage activation (profibrotic macrophages activation) with characteristic expression of cell surface markers of CD206, CD204 CD163, Col1a1, Col3a1. On the other hand, rIFN-γ stimulated iNOS expression was not altered with null mutation of CHIT1. These studies strongly support a notion that CHIT1 contributes to the development of profibrotic, alternative macrophage activation, but not with classical activation. However, the specific mechanism(s) or pathways that CHIT1 uses to regulate macrophage activation remains to be determined.

#### CHIT1 in Fibroblasts Proliferation and Myofibroblast Transformation

Fibroblasts are the major effector cells responsible for fibrotic tissue responses in that TGF-β1 plays an essential role. In *in vitro* studies using normal human lung fibroblasts (NHLF), CHIT1 enhanced TGF-β1-stimulated fibroblast proliferation and myofibroblasts transformation while CHIT1 itself did not significantly alter at fibroblasts proliferation (Lee et al., 2012; Lee et al., 2019). Similarly, CHIT1 itself did not alter fibroblast differentiation into myofibroblasts as assessed by α-smooth muscle actin (α-SMA) expression but did augment the ability of TGF-β1 to enhance α-SMA accumulation (Lee et al., 2019). This result suggests that CHIT1 enhances TGF-β1-stimulated fibroblast proliferation and myofibroblast transformation.

#### CHIT1 in TGF-β-Stimulated Pulmonary Fibrosis and its Signaling

Studies employing WT and CHIT1 null mutant mice demonstrated that the ability of TGF-β1 to stimulate ECM proteins including fibronectin, type 1 collagen, and other ECM molecules in a CHIT1 dependent manner (Lee et al., 2012). On the other hand, the exaggerated fibrotic responses were noted in lungs from the mice in which CHIT1 and TGF-β1 were simultaneously expressed compared to the mice in which each was expressed individually (Lee et al., 2019). They demonstrated that transgenic TGF-β1 increased canonical Smad2/3 and noncanonical MAPK/Erk, and Akt activation compared to wild type control mice, and these TGF-β stimulated signaling was further enhanced in lungs of CHIT1 and TGF-β1 double transgenic mice (Lee et al., 2019). These studies suggest that CHIT1 is required and sufficient for the development of pulmonary fibrosis in that TGF-β1 plays a critical role.

#### CHIT1 Interacts With Tgfbrap1 and FoxO3 as a Mechanism to Enhance TGF-β Signaling

Since CHIT1 can be found in both extracellular and intracellular compartments, it is reasonable to assume specific receptors or interacting partners are mediating CHIT1 effects in the lung. On this regard, potential CHIT1 binding partners were first defined using a yeast 2 hybrid (Y2H) screening assay with a lung cDNA library (Lee et al., 2019). This approach identified transforming growth factor receptor beta associate protein 1 (Tgfbrap1) and forkhead box O3 (FoxO3) as CHIT1-interacting proteins. Tgfbrap1 has been reported to be a chaperone for Smad signaling (Wurthner et al., 2001) and FoxO3 is a transcription factor for multiple genes that play critical roles in metabolism, cellular stress response, tissue remodeling, and disease progression (Nho and Hergert, 2014; Webb et al., 2016). Recently, FoxO3 was reported as a key regulator of pulmonary fibrosis (Al-Tamari et al., 2018). The co-immunoprecipitation (Co-IP) and immunoblot (IB) assay and double immunohistochemistry (IHC) evaluations of cells transfected with CHIT1, Tgfbrap1 and FoxO3 demonstrated significant molecular interactions between CHIT1 and Tgfbrap1 or FoxO3 (Lee et al., 2019). In these evaluations, it is interesting to note that the CHIT1 negatively regulates the expression of Smad7, one of the inhibitor smads, through interaction with FoxO3, that potentially provide positive feedback loop to further enhance TGF-β signaling and effector fucntion through CHIT-Tgfbrap1 interactions.

**FIGURE 2:**
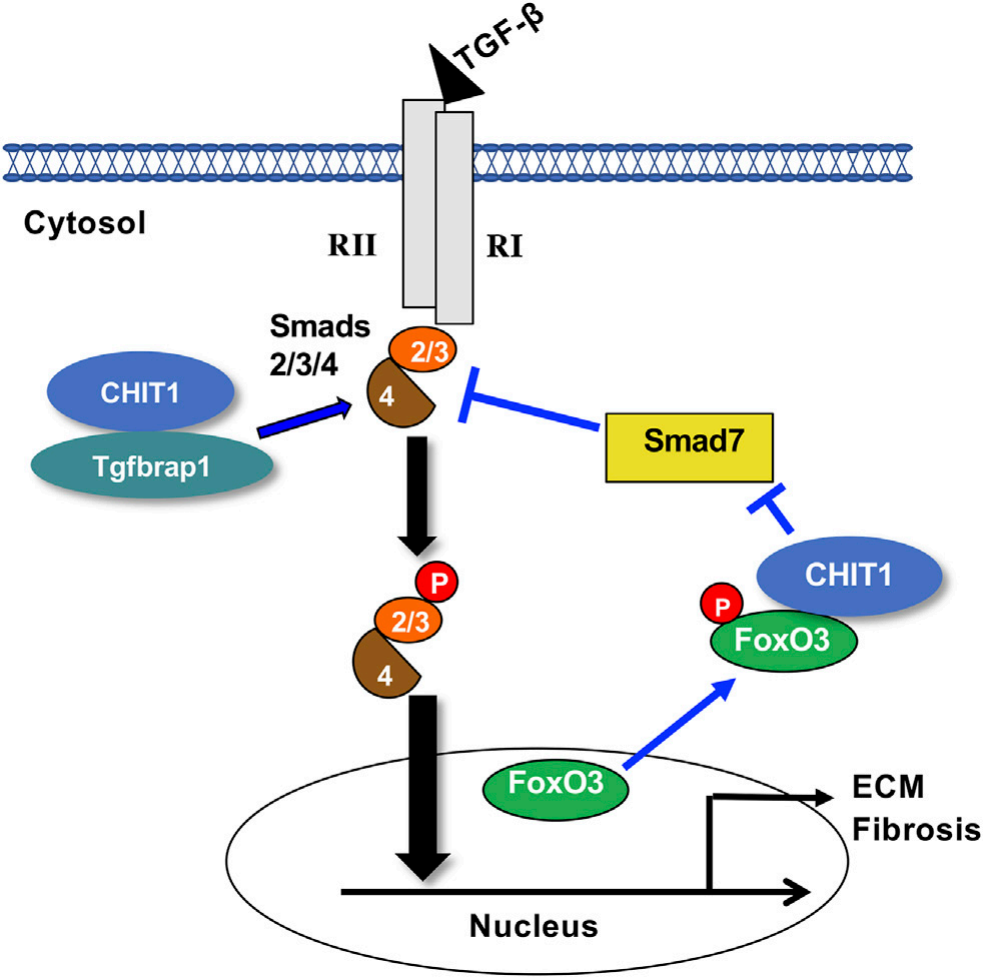
Suggested pathway that chitinase 1 uses to regulate TGF-β signaling. Chitinase 1 (CHIT1) binds with transforming growth factor beta receptor associated protein 1 (Tgfbrap1), enhances the canonical receptor mediated Smads2/3 signaling potentially through recruiting of Co-Smad4. CHIT1 also interacts with transcription factor forkhead box O3 (FoxO3) that reduces the nuclear FoxO3 and that results into decreased the Smad7 expression. RI, TGF-β receptor 1; RII, TGF-β receptor 2; p, phosphorylation; ECM, extracellular matrix proteins.

In summary, recent studies strongly support an important role of CHIT1 in the pathogenesis of pulmonary fibrosis *via* modulation of TGF-β1 signaling and effector function. They also highlight that CHIT1 uses Tgfbrap1 and FoxO3 as interacting partners to regulate canonical and non-canonical TGF-β1 signaling and Smad7 expression as schematically illustrated in Figure 2. These studies provide strong supportive evidence for introducing CHIT1 inhibitor(s) as a novel therapeutic drug for pulmonary fibrosis. However, details of protein-protein interactions between CHIT1 and its interacting partners and their specific role in profibrotic macrophage and fibroblasts activation remain to be determined.

### Role and Mechanism of CHI3L1 in the Regulation of Pulmonary Fibrosis

CHI3L1 (also referred to as YKL-40 in human and Chil1/BRP-39 in mice (Lee and Elias, 2010)), a prototype of chitinase-like proteins, binds to chitin but does not cleave it (Lee et al., 2009). The importance of CHI3L1 can be readily appreciated in the diseases characterized by inflammation and remodeling in which CHI3L1 excess has been documented (Recklies et al., 2002; Sohn et al., 2010; Areshkov et al., 2012; Dela Cruz et al., 2012). In many of these disorders, CHI3L1 is likely produced as a protective response based on its ability to simultaneously decrease epithelial cell apoptosis while stimulating fibroproliferative repair. In patients with IPF, high serum and lung levels of CHI3L1 can be detected and are associated with poor survival (Korthagen et al., 2011; Lee et al., 2011). These findings support the major role of CHI3L1 in fibroproliferative responses including pulmonary fibrosis. However, the roles of CHI3L1 in these diseases have not been fully elucidated because the biologic functions of CHI3L1 and the mechanisms by which they are mediated have only recently begun to be studied.

#### CHI3L1 Expression in Human Lung Fibrosis

CHI3L1 is implicated as a serum biomarker in diseases with fibrosis, inflammation, and tissue remodeling. In IPF, serum and BALF CHI3L1 levels are significantly higher in IPF patients than in controls, and increased CHI3L1 expression was observed in alveolar macrophages and bronchiolar epithelial cells adjacent to fibrotic lesions (Furuhashi et al., 2010). Importantly, the circulatory levels of CHI3L1 are inversely correlated with prognosis of the patients. Recent epidemiologic studies including 85 patients with IPF, and 126 controls demonstrated that high serum and BALF CHI3L1 levels are associated with poor survival: IPF patients with high serum or high BALF CHI3L1 levels had significantly shorter survival than those with low CHI3L1 levels in serum or BALF (Korthagen et al., 2011). Notably, in patients with both low serum and low BALF CHI3L1 levels, no IPF related mortality was observed, suggesting that serum CHI3L1 could be a useful prognostic marker (Korthagen et al., 2011). Consistent with these reports, a significant increase in both quantities and percentages of CHI3L1-expresssing macrophages and epithelial cells in IPF lungs. In addition, in a cohort of 64 IPF patients and 42 age-matched controls, CHI3L1 levels were elevated in patients with IPF, and high levels of CHI3L1 are associated with severe disease progression as defined by lung transplantation or death (Zhou et al., 2014). Since then, numerus studies have showed that the serum CHI3L1 levels are increased in patients with other types of ILD, including connective tissue-related ILD (Hozumi et al., 2017; Furukawa et al., 2019; Jiang et al., 2019), sarcoidosis (Johansen et al., 2005; Kruit et al., 2007), cryptogenic tissue pneumonia (Korthagen et al., 2014; Long et al., 2017), asbestosis-ILD (Väänänen et al., 2017), and idiopathic nonspecific interstitial (Korthagen et al., 2014; Long et al., 2017).

#### CHI3L1 and its Receptors

Because CHI3L1 lacks enzymatic activity, recent studies were undertaken to identify if CHI3L1 mediates its effector functions through novel cell surface receptors. Yeast Two Hybrid (Y2H) screening and other binding and cellular approaches demonstrate that CHI3L1 binds to, signals and confers tissue responses *via* at least two receptor complexes: IL-13Rα2 (and its beta subunit transmembrane protein 219 (TMEM 219)) and CRTH2 (Chemoattractant Receptor-homologous molecule expressed on Th2 cells) (He et al., 2013; Zhou et al., 2015). These studies further revealed that IL-13Rα2/TMEM219 is expressed on lung epithelial cells and mediates CHI3L1’s protective effects against cell death. On the other hand, CHI3L1 interacts with CRTH2 and promotes fibrosis *via* a mechanism that involves fibroblast proliferation and matrix deposition.

CRTH2 is a G-protein coupled receptor that binds with Prostaglandin D2 (PGD2). The interactions between CHI3L1 and CRTH2 have been further verified with co-Immunoprecipitation and immunoblot assay and co-localization immunohistochemistry (IHC) (Zhou et al., 2015). CRTH2 played an important role in augmenting CHI3L1-medaited fibroproliferative responses because collagen accumulation and extracellular gene expression were significantly reduced in the mice lacking CRTH2 or in mice treated with a CRTH2 inhibitor (Cay10471) (Zhou et al., 2015). Consistent with these findings, a recent study indicated that CRTH2 contributed to the development of fibrosis in kidney (Ito et al., 2012).

Gal-3 is a β-galactoside–binding lectin with pro-fibrotic effects (Young et al., 2006; Cullinane et al., 2014; Li et al., 2014). Further studies demonstrate that Gal-3 interferes with CHI3L1 signaling by competing for IL-13Rα2 binding. As a result, Gal-3 diminishes the anti-apoptotic effects of CHI3L1 in epithelial cells while increasing macrophage Wnt/ß-catenin signaling (Zhou et al., 2018). Therefore, Gal-3 contributes to the exaggerated injury and fibroproliferative repair response by altering the anti-apoptotic and fibroproliferative effects of CHI3L1 and its receptors.

Geng et al. demonstrated that CHI3L1 physically interacts with CD44, a cell-surface transmembrane glycoprotein involved in cell growth, survival, and differentiation. They elegantly showed that the interaction between CHI3L1 and CD44 activated the Erk and Akt pathways, along with phosphorylation of β-catenin (Geng et al., 2018). Other potential CHI3L1 receptors/binding partners include heparin and heparin-like molecules (Ngernyuang et al., 2018), and hyaluronic acid (Nishikawa and Millis, 2003; Zheng et al., 2005). However, the specific roles of CD44, heparin-like molecules, and hyaluronic acid and their interactions with CHI3L1 in the pathogenesis of pulmonary fibrosis remain to be determined.

#### CHI3L1 and cells Implicated in the Pathogenesis of Pulmonary Fibrosis

Abundant data indicate that profibrotic macrophages (traditionally called as M2 alternatively activated macrophages) play an important role in the development of lung fibrosis. Macrophages remain as a major cellular source of CHI3L1 production in IPF lungs (Furuhashi et al., 2010; Zhou et al., 2014). Murine bleomycin lung fibrosis model showed a predominant shift toward alternative macrophage activation in CHI3L1 overexpressing Tg mice, and eradication of these profibrotic macrophages is sufficient to reduce tissue fibrosis (Zhou et al., 2014). Consistently, CD206^+^ alternatively activated, profibrotic macrophages are increased in the IPF lung, and circulating monocytes of IPF patients exhibit increased expression of alternative activation marker CD206 (Zhou et al., 2014).

The role of CHI3L1 on fibroblast proliferation/differentiation was explored using a three-dimensional cell culture model (Zhou et al., 2014). These studies demonstrated that, in contrast to unstimulated cells, fibroblasts grown in the presence of recombinant CHI3L1 showed increased cell density, and increased proliferation. Additionally, the addition of CHI3L1 was sufficient to modestly induce α-SMA detection in MRC5 cells cultured in this model, and a contractile fibroblast phenotype was observed as shown by the shrinking and rupture of the matrix slices (Zhou et al., 2014). It was reported that the CD44, a putative receptor for CHI3L1 (Cohen et al., 2017), mediates invasive fibroblasts phenotypes leading to severe lung fibrosis (Li et al., 2011). Recent studies reported that CHI3L1 induces the expression of PD-L1 in various lung cells (Ma et al., 2021) and the increased expression of PD-L1 is a major characteristic of transformed and invasive fibroblasts (Geng et al., 2019). These findings show that CHI3L1 directly regulates fibroblast proliferation, and to a smaller extent, myofibroblast transformation with invasive nature, thereby demonstrating a role for CHI3L1 in the regulation of all three phases of the fibroblast-mediated fibrotic responses.

The role of Gal-3 and its interactions with CHI3L1 and/or its receptor components in fibroproliferative repair response was explored in macrophages and fibroblasts. These studies demonstrate that Gal-3 is up-regulated in murine models of pulmonary fibrosis. Interestingly, these studies demonstrated spatial differences in its cellular and tissue effects: Extracellular Gal-3 drives epithelial apoptosis when in the extracellular space while intracellular accumulation of Gal3 in fibroblasts and macrophages stimulates fibroblast proliferation, myofibroblast differentiation and profibrotic macrophage differentiation (Zhou et al., 2018).

#### CHI3L1 and its Receptors in Hermansky-Pudlak Syndrome-Associated Lung Fibrosis

Hermansky-Pudlack Syndrome (HPS) is a rare, genetic, multisystem disorder characterized by oculocutaneous albinism (OCA), bleeding diathesis, immunodeficiency, granulomatous colitis, and pulmonary fibrosis (Gahl and Huizing, 2012). Ten genetic subtypes (HPS1-10) have been described with each mutation affecting the function of lysosome-related organelles (LROs) (Schinella et al., 1980; Anderson et al., 2003). The dysfunction of melanosomes accounts for the oculocutaneous albinism and visual impairment found in all HPS patients ((Gahl et al., 1998)). The dysfunction of platelet dense granules accounts for the bleeding disorder that is often the presenting complaint of the disease ((Hermansky and Pudlak, 1959; Gahl et al., 1998)). Ceroid deposition also occurs in multiple organs, and inflammatory bowel disease has been reported in various subtypes of HPS (Schinella et al., 1980; Mahadeo et al., 1991; Parker et al., 1997; Tsilou et al., 2004). Pulmonary Fibrosis has been appreciated in HPS-1 and HPS-4 patients, whose genetic defects are in biogenesis of lysosome-related organelle complex 3 (BLOC-3), which includes HPS1 and HPS4 proteins, and, less commonly, HPS-2 patients (Brantly et al., 2000; Anderson et al., 2003; Chiang et al., 2003; Li et al., 2004; Carmona-Rivera et al., 2013). Due to the untreatable and progressive nature of the pulmonary fibrosis of HPS, this complication is the leading cause of death (Pierson et al., 2006). However, there is no way to predict in which HPS-1 or HPS-4 patients are at risk for lung disease, or which patients will progress most rapidly. In addition, although it is known that murine genetic models of HPS-1 manifest exaggerated injury and fibroproliferative repair responses to fibrogenic agents like bleomycin (Young et al., 2007), the mechanism(s) by which LRO-related defects in trafficking lead to injury and fibrosis have not been adequately defined.

Zhou et al. found that in HPS, levels of CHI3L1 are higher in patients with HPS-PF in comparison with patients without pulmonary fibrosis, where higher levels are associated with greater disease severity (Zhou et al., 2015). In murine models, the animals with BLOC-3 mutation have a defect in the ability of CHI3L1 to restrain epithelial cell death, yet CHI3L1 exhibits exaggerated fibroproliferative effects, promoting fibrosis by inducing alternative macrophage activation and fibroblast proliferation (Zhou et al., 2015). The two distinctive features of CHI3L1 are mediated by trafficking of two CHI3LI receptors, IL-13Rα2 and CRTH2. The increase of apoptosis results from the abnormal localization of IL-13Rα2, which is caused by the dysfunction of BLOC-3. Fibrotic effects were caused by interactions between CHI3L1 and CRTH2 receptors which traffic normally (Zhou et al., 2015). These studies suggest that CHI3L1 and its receptors are dysregulated and play critical roles in the generation and progression of lung fibrosis associated with HPS. In addition, these responses are largely mediated by CRTH2, which may serve as a therapeutic target. Multiple clinical trials were designed to assess the effects of CRTH2 antagonism on asthma control. Future studies will be required to explore the possibility of repurposing these small molecular CRTH2 antagonists for HPS-PF treatment.

Strong evidence has indicated a critical role of Gal-3 in the development of HPS-PF. In samples from HPS-1 patients, AT2 cells, alveolar macrophages, and fibroblasts have high levels of Gal-3 expression and intracellular accumulation. It is speculated that the accumulation of Gal-3 in the cells of HPS individuals can be explained by the abnormal trafficking in the endosomal recycling compartment, which can contribute to fibrogenesis in HPS-PF (Cullinane et al., 2014). Consistently, murine studies have found that Gal-3 has increased levels in the extracellular space, traffics abnormally, and accumulates in lung fibroblasts and macrophages. Extracellular Gal-3 stimulates epithelial apoptosis and intracellular Gal-3 enhances fibroblast survival and proliferation as well as myofibroblast and macrophage differentiation. It can be speculated that Gal-3-based therapies may very well act in an additive or synergistic manner with interventions that augment membrane expression of IL-13Rα2 or block CRTH2. Additional investigations will be required to assess the utility of each of these approaches.

### CHIT1/CHI3L1 in Collagen Stability and Bioactivity

As discussed above, ChIT1 and CHI3L1 distinctly contribute to pulmonary fibrosis using unique receptors or interacting molecules and signaling pathways. However, CHIT1 and CHI3L1 also have similar regulatory functions in the development of profibrotic macrophage activation, fibroblasts proliferation and myofibroblasts differentiation, the major hallmarks of pulmonary fibrosis. These studies suggest a possibility that CHIT1 and CHI3L1 can share specific biologic function that are independent of their receptors or interacting partners. Both CHIT1 and CHI3L1, as members of 18 Glycosyl hydrolase family, contain carbohydrate binding domain or motif (CBD or CBM) that has a binding ability with various forms of carbohydrates including chitin, hyaluronan and collagens (Crasson et al., 2017). Studies also suggest that the CBD is a critical region determining the biologic activity of CHIT1 and CHI3L1 (Chen et al., 2011; Crasson et al., 2017). However, the details of physical binding between CHIT1 or CHI3L1 with collagens through CBD and its effect on the profibrotic macrophage activation or myofibroblast transformation remain to be determined.

### CHIT1/CHI3L1 as Modifiers of TGF-β Expression and its Signaling

As discussed earlier, TGF-β is a potent profibrotic cytokine that mediates pulmonary fibrosis especially associated with profibrotic activity of CHIT1. It is interesting to note that CHI3L1 was also reported to increase the expression of TGF-β and its signaling in the lung (He et al., 2013). This means that both CHIT1 and CHI3L1 could modulate the effector function of TGF-β possibly through synergistic fashion where these moieties are co-expressed. However, the interactions between these two in the pathogenesis of pulmonary fibrosis has not been determined. Since other genes, such as Wilms’ tumor 1 (WT-1) or transforming growth factor alpha (TGF-α), also significantly regulate the expression and or signaling of TGF-β directly or indirectly, indued fibrotic tissue responses especailly in distal areas of lung (Madala et al., 2014; Sontake et al., 2018). If this is the case, whether and how the CHIT1/CHI3L1 axis is implicated in the development both parenchymal and subpleural fibrosis by activating TGF-β signaling would be an interesting question to be determined.

### CHIT1/CHI3L1 in Heterocellular Crosstalk in Progressive Pulmonary Fibrosis

**FIGURE 3:**
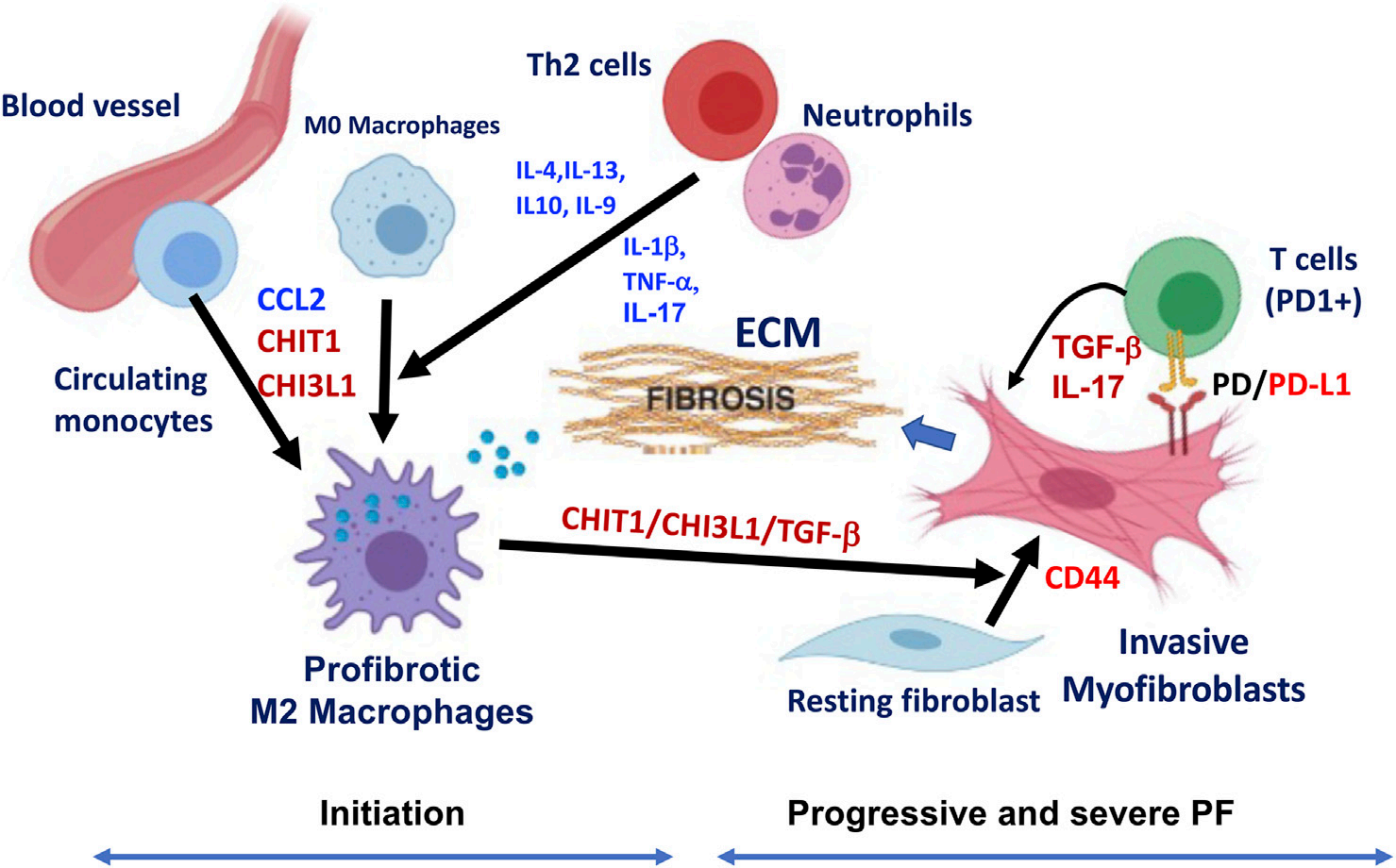
Schematic illustration of hypothetical pathway leading to the development of profibrotic macrophages and invasive fibroblasts in the pathogenesis of pulmonary fibrosis (PF). Although multiple cells and mediators are involved in this process, implication of CHIT1/CHI3L1 and PD-1/PD-L1 axis are highlighted as major players in the development of profibrotic macrophages and invasive fibroblasts/myofibroblasts leading to progressive and severe pulmonary fibrosis through suggested heterocellular interactions. As indicated, fibrotic macrophages are recruited from the circulatory monocytes potentially through CCL2, CHIT1 or CHI3L1 then develops to alternatively activated profibrotic macrophages that highly expressing CHIT1, CHI3L1 and TGF-β. These mediators synergistically interact to transform resting fibroblasts to myofibroblasts with invasive nature through CD44. PD-L1 expressing invasive fibroblasts interact with PD-1 (+) T cells that further induce the profibrotic mediators including TGF-β and IL-17, that resulted into fibrotic tissue response with excessive extracellular matrix (ECM) accumulation. Th2 cytokines (IL-4, IL-13, IL-10, and IL-9) and proinflammatory cytokines (IL-1β, TNF-α, and IL-17) from TH2 cells and neutrophils can also contribute to profibrotic macrophage activation and subsequent fibroblasts proliferation and activation leading to pulmonary fibrosis (*The image is created with* *BioRender.com* ).

As discussed before, IPF is a disease characterized by the excessive accumulation of extracellular matrix (ECM) in the lung parenchyma with an unchecked, vicious cycle of repeated injury and abnormal repair responses. Thus, it is reasonable to speculate that there could be a temporal association between the emergence of profibrotic macrophages and invasive fibroblasts during the progression of pulmonary fibrosis and a potential positive feedback loop to generate a pathologic progressive fibrosis. If this is the case, the heterocellular crosstalk between “profibrotic macrophages” and “invasive fibroblasts” could be the major component of the profibrotic microenvironment responsible for progressive pulmonary fibrosis. In addition, the PD-1/PD-L1 axis is another fibrosis regulatory mechanism that consists of a positive feedback loop between PD-L1^+^ invasive fibroblasts and PD-1^+^CD4^+^ T cells and contributes to progressive fibrosis through enhanced expression of TGF-β and IL-17 (Celada et al., 2018). Since CHIT1 and CHI3L1 play an essential role in these processes, targeting CHIT1 or CHI3L1 or both would be an effective therapeutic strategy that potentially blocks or reverses ongoing fibrosis as schematically illustrated in Figure 3.

## Summary

Recently, increasing number of studies are demonstrating that chitinase and chitinase like proteins (C/CLPs) represented by CHIT1 and CHI3L1 are significantly implicated in the pathogenesis of various human diseases including pulmonary fibrosis. However, the C/CLPs biology underlying specific pathologic process of the diseases has not been fully understood. A substantial body of literatures are strongly support that both CHIT1 and CHI3L1 contribute to the development and progression of pulmonary fibrosis through regulation of profibrotic macrophage activation and/or invasive myofibroblast differentiation. However, no data are currently available to support whether CHIT1 and CHI3L1 regulation of fibroproliferative cellular and tissue responses occurs in an independent manner or dependently each other. Since CHI3L1 does not have enzyme activity while retaining binding capacity with same substrates that CHIT1 uses, we speculate that CBD, not catalytic domain, is likely to function as a major domain responsible for profibrotic activities. Thus, development of antifibrotic therapeutics against CBD would be a reasonable strategy to block bioactivities of CHIT1 and CHI3L1. In addition, further mechanistic understanding on the putative receptors and interacting partners of CHIT1 and CHI3L1 in profibrotic macrophage activation and invasive fibroblast differentiation and their heterocellular interactions will be crucial for the development of effective therapeutics targeting CHIT1 and CHI3L1 for the patients with progressive pulmonary fibrosis.
